# Burning Questions: Oral Ulceration Secondary to Improper Bisphosphonate Administration in Advanced Dementia

**DOI:** 10.3390/geriatrics11050128

**Published:** 2026-09-08

**Authors:** Kate Murphy, Eleanor Marks, Kieran O’Connor, Elizabeth Moloney

**Affiliations:** Department of Geriatric Medicine, Mercy University Hospital, T12 WE28 Cork City, Ireland

**Keywords:** oral care, oral ulcer, bisphosphonate, dementia, dysphagia, older adult

## Abstract

**Background:** Oral bisphosphonates are first-line osteoporosis therapy but can cause mucosal chemical injury when tablets are retained rather than swallowed correctly. This risk is heightened in cognitively impaired older adults with dysphagia or difficulty complying with medication administration guidelines. **Case Presentation:** A 73-year-old female nursing home resident with advanced vascular dementia presented with 24 h of oral pain, reduced intake, lethargy and new facial swelling. She had received monthly ibandronic acid 150 mg for two years, with documented difficulty swallowing tablets but no formal swallowing assessment. Examination showed ulceration of the left lower lip and anterior tongue with mild vestibular swelling. Clinical investigations excluded haematinic deficiency and osteonecrosis; traumatic, lichenoid, vesiculobullous and neoplastic causes were considered unlikely based on distribution, clinical features and temporal association with the recent dose. Bisphosphonate-associated chemical mucosal injury was diagnosed by hospital dental services. Ibandronic acid was discontinued, topical treatment initiated, and ulcers resolved by three weeks. Denosumab was substituted in view of the patient’s dysphagia and cognitive status. **Discussion:** This case supports the existing literature identifying inappropriate administration—often linked to dysphagia or physical impairment—as a leading cause of oral bisphosphonate-associated ulceration, typically affecting the tongue and lower lip. Ibandronate-related cases are rarely reported, likely reflecting prescribing frequency rather than lower risk. Current guidelines lack explicit recommendations addressing dysphagia or cognition in prescribing decisions. **Conclusions:** Presence of dysphagia and cognitive status should be assessed before and during oral bisphosphonate therapy. New oral pain, ulceration or facial swelling should prompt oral examination and medication review, with alternative antiresorptive agents considered when oral administration is impaired.

## 1. Introduction

Bisphosphonates are the cornerstone of treatment of osteoporosis, with well-established efficacy in reducing fracture risk, particularly in older populations [1]. However, their use is associated with adverse effects, including oesophageal ulceration and osteonecrosis of the jaw [2]. Less widely recognised is direct injury to the oral mucosa. Nitrogen-containing bisphosphonates are strongly acidic and chemically caustic in solution; when a tablet is retained in the mouth rather than swallowed promptly with adequate water, prolonged contact produces a chemical burn of the epithelium [3]. Local inhibition of farnesyl pyrophosphate synthase within keratinocytes is thought to compound this insult by impairing epithelial turnover and delaying repair so that a superficial injury evolves into a persistent ulcer [4]. Correct administration—an upright posture, a full glass of plain water, and no recumbency or oral intake for the following 60 min—is therefore critical to mitigating these risks [5].

We present a case of bisphosphonate-induced oral ulceration in a patient with advanced dementia, underscoring the risks associated with impaired administration and highlighting the need for tailored prescribing practices in cognitively impaired populations.

## 2. Case History

A 73-year-old female nursing home resident was referred to an Irish emergency department with acute oral pain, reduced oral intake and lethargy for 24 h. She had been a resident in the nursing home for two years. Relevant past medical history included advanced vascular dementia, epilepsy and hypothyroidism. She was severely cognitively impaired, with limited verbal fluency. She required full assistance with activities of daily living (washing, dressing, feeding, toileting). Baseline mobility status was supervision with a walking aid. Nursing staff noted the resident had variable engagement with oral care routines. Oral bisphosphonate therapy had been commenced 2 years previously following a diagnosis of osteoporosis on DEXA imaging. She had been prescribed monthly oral ibandronic acid 150 mg for the preceding two years and had taken the most recent dose forty-eight hours prior to symptom onset. Concurrent medication comprised aspirin, bisoprolol, escitalopram, levetiracetam, carbamazepine, eltroxin, triazolam and quetiapine. There was no documented history of methotrexate, nicorandil or regular non-steroidal anti-inflammatory use.

Nursing staff noted frequent resistance to taking medications and difficulty swallowing tablets. A formal swallowing assessment by speech and language therapy services had not occurred during the previous two years. A recorded weight was not documented in nursing transfer or hospital medical notes. The patient was edentulous and did not wear full dentures. She was not known to have xerostomia, although several of her regular medications, including escitalopram, quetiapine and triazolam, carry recognised xerostomic potential. Nursing home staff contacted a family member the day of transfer to the emergency department to advise they had noted a new left-sided facial swelling.

### 2.1. Clinical Examination

**Figure 1:**
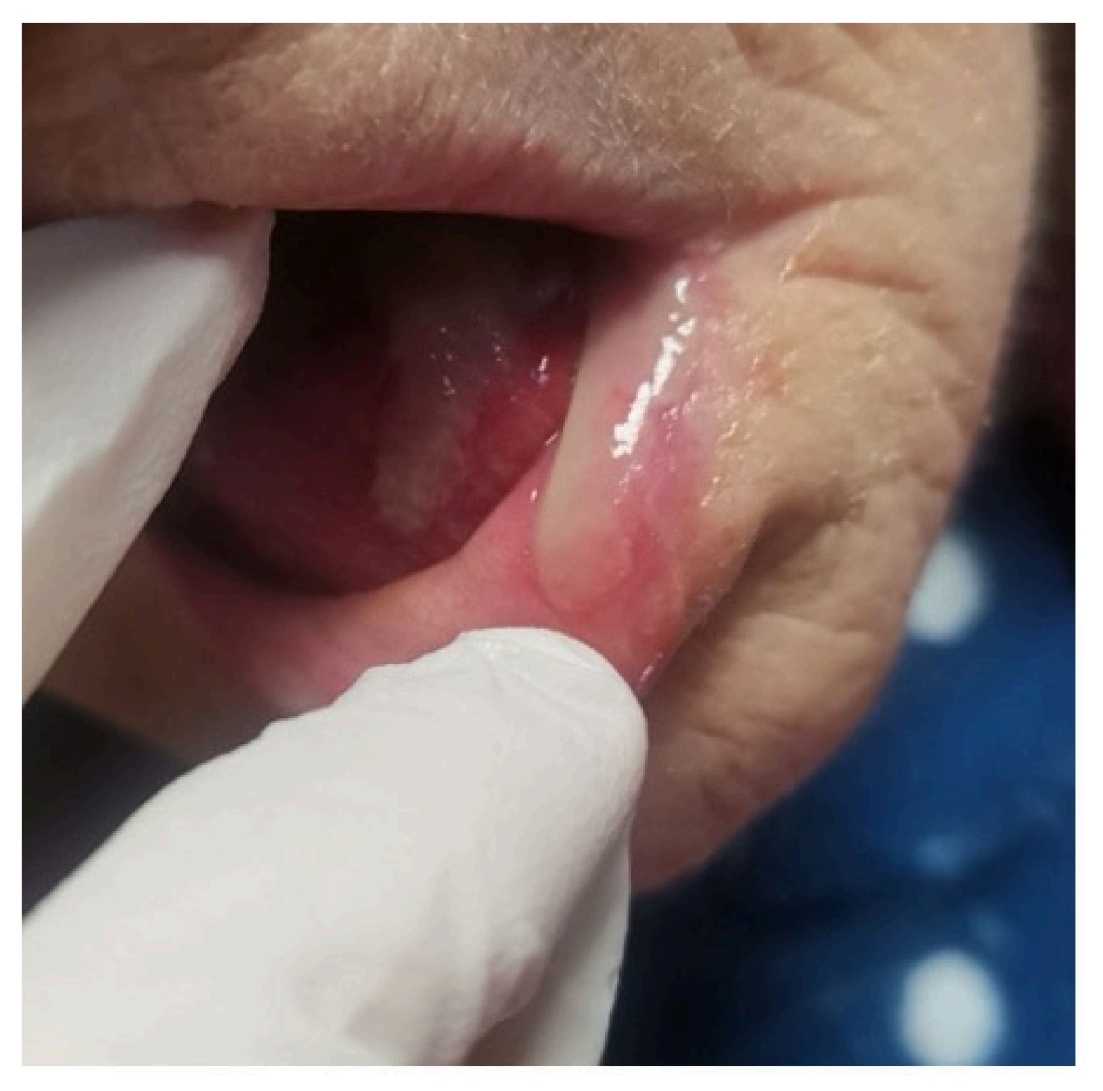
Left lower lip ulcer at presentation.

**Figure 2:**
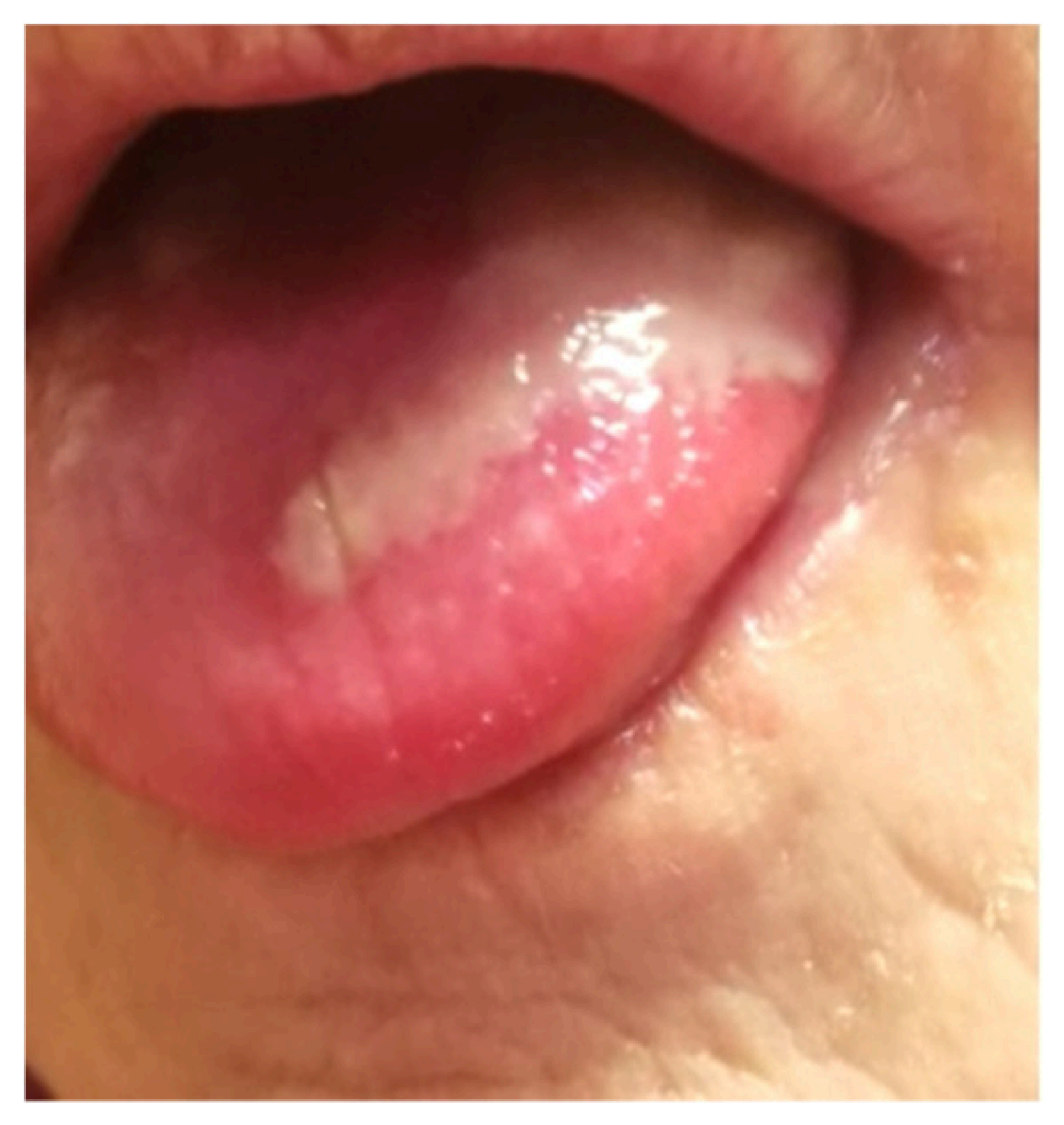
Lateral tongue border ulcer at presentation.

Clinical examination in the emergency department revealed two areas of mucosal ulceration: one involving the left lower lip and another on the left anterior tongue (Figure 1 and Figure 2). Mild left lower lip and oral vestibule swelling was present.

The patient indicated she had a painful mouth and was initially reluctant to allow oral examination. No retained food, tablets or retained root fragments were identified. There was no cervical lymphadenopathy, and no extraoral mucosal ulceration or other mucosal involvement was noted. Vital signs were within normal parameters.

### 2.2. Diagnostic Assessment and Management

Blood results within the previous three months did not indicate anaemia or haematinic deficiency. Plain film X-ray imaging of the jaw demonstrated no evidence of mandibular osteonecrosis, sequestration or other bony abnormality. Assessment was directed at excluding infective, traumatic and neoplastic causes of oral ulceration and whether medication administration had contributed to the lesions. The clinical differential for acute oral ulceration in a frail older adult is broad, and the diagnosis in this case was one of exclusion, supported by a clear temporal relationship to drug administration and by resolution on withdrawal.

Traumatic ulceration was the leading alternative diagnosis, given the patient’s cognitive impairment, dependence for feeding and oral care, and potential for inadvertent mechanical trauma. However, no obvious traumatic source was identified, and the distribution of the lesions, together with the temporal relationship to the recent ibandronate dose, favoured a medication-related process.

Medication-related mucosal disease was considered given the patient’s medication regime. However, there had been no recent medication changes, and there was no documented history of recurrent ulceration associated with her regular medications. A drug-related lichenoid reaction was considered less likely because there were no characteristic reticular mucosal changes.

Vesiculobullous disease, including mucous membrane pemphigoid, pemphigus vulgaris and erosive lichen planus, characteristically produces multifocal, chronic or relapsing disease with desquamative gingivitis and frequently extraoral involvement. This diagnosis was considered unlikely because no vesicles or bullae were identified and there was no involvement of other oral or extraoral mucosal surfaces, and the clinical course was inconsistent with chronic vesiculobullous disease.

Oral malignancy, principally squamous cell carcinoma, was considered because persistent ulceration of the tongue or lip warrants appropriate clinical vigilance. However, the acute onset, absence of induration or cervical lymphadenopathy, temporal relationship with ibandronate administration and complete clinical resolution within three weeks made malignancy unlikely. Biopsy was not undertaken due to the rapid resolution. Persistent or recurrent ulceration would have warranted further investigation, including biopsy where clinically indicated.

Following review by the hospital dental service, the clinical diagnosis was bisphosphonate-associated chemical mucosal injury, most likely resulting from prolonged contact between ibandronic acid and the oral mucosa. The presumed mechanism was retention of the tablet in the setting of advanced cognitive impairment and probable oropharyngeal dysfunction.

The patient had a short inpatient admission under a geriatric medicine team. Ibandronic acid was discontinued. Symptomatic management consisted of benzydamine hydrochloride spray three times a day for two weeks and topical lidocaine-containing mouthwash twice a day for two weeks. At three-week follow-up, the ulcers had resolved, and the patient had resumed an adequate oral intake. No recurrent lesions were reported.

The geriatric medicine team undertook an osteoporosis medication review and implemented an alternative antiresorptive strategy. Denosumab was chosen as the alternative agent, taking into account the patients’ cognitive status, swallowing ability and ability to comply with administration requirements.

## 3. Discussion

Oral bisphosphonate-associated mucosal ulceration is an uncommon but clinically important adverse effect. It is particularly relevant in older adults who have cognitive impairment, dysphagia or dependence on carers for medication administration.

A scoping review by Psimma et al. [6] identified 56 reported cases of oral adverse events associated with oral bisphosphonates, comprising 22 case reports, one case series and three reviews. Oral ulceration was the predominant presentation, accounting for approximately 80% of reported mucosal adverse events. Eighty-eight percent of reported patients were women; however, this should not be interpreted as evidence of a biological female predisposition to bisphosphonate-induced mucosal ulceration. The apparent predominance may reflect the epidemiology of osteoporosis and the greater use of oral bisphosphonates among older women [7]. Importantly, inappropriate administration was considered the most likely cause in more than half of cases, with physical impairment and dysphagia specifically identified as contributors to incorrect administration. The tongue and lower lip were among the most frequently affected sites.

McLean et al. have similarly described severe oral ulceration following incorrect use of alendronate [8], and Gonzalez-Moles reported patients experiencing chemical burns due to retaining tablets in the mouth [9].

The present case shares several features with these reports. The patient was an older woman with substantial cognitive impairment, difficulty swallowing tablets and dependence on carers for medication administration. She had received a monthly oral bisphosphonate and developed painful oral ulceration shortly after her most recent dose. The location of the lesions on the lower lip and tongue is also consistent with the distribution reported in the published literature.

The present case differs from many previously reported cases in that the patient was receiving ibandronic acid rather than alendronic acid. The relative paucity of reported ibandronate-associated oral mucosal lesions may reflect the less frequent use of this preparation rather than an absence of risk. Importantly, the current ibandronate product information explicitly states that tablets should be swallowed whole and should not be chewed or sucked because of the potential for oropharyngeal ulceration [10]. It also recommends that patients remain upright for at least 60 min and advises caution in patients with dysphagia or other upper gastrointestinal disorders. There is insufficient evidence to establish reliable recurrence for oral bisphosphonate-associated ulceration, as the published literature consists predominantly of isolated case reports and small case series [11]. Clinical review until complete healing is appropriate, particularly when ulceration is associated with pain, dysphagia or reduced oral intake. However, persistent, recurrent or indurated ulceration should prompt reassessment and consideration of further investigation.

The United Kingdom and European osteoporosis guidelines offer general advice to consider alternative treatment options if first-line therapies are unsuitable or not tolerated regarding gastrointestinal side effects, including intravenous therapies or denosumab [5,12]. However, neither European nor international guidelines explicitly address swallowing impairment, cognition, or posture in prescribing decisions, meaning there is a gap in the osteoporosis management literature. Oral care researchers have emphasised the crucial role of multidisciplinary collaboration in addressing the oral and swallowing health needs of older adults in care homes. Recent multinational analyses show that coordinated management involving physicians, dentists, speech and language therapists, and nursing staff improves outcomes where oral and systemic health intersect [13]. Published guidance from the UK Royal College of Speech and Language Therapy highlights various signs and symptoms which suggest a person is having swallowing difficulties, such as coughing or throat clearing after eating or drinking, food staying in the mouth after chewing and declining food and drink [14]. If noted, the advice is to refer the person for speech and language assessment.

The Health Service Executive (HSE) in Ireland provides public health and social care services, manages hospitals and community care, and implements national health policies. In 2025, the HSE published a guidance document, titled “Supporting Smiles”, on provision of oral care for adults in acute and community care facilities with dementia-specific oral care guidance [15]. This document is supported by a national staff training programme, led by dental hygienist “champions” in regional centres. UK approaches based on NICE guidance have incorporated oral-health assessment, individualised oral-care plans, staff training, designated oral-health champions and access to dental services [16]. Australian residential care initiatives have similarly explored structured oral-health assessment and nurse-led individualised oral care planning following staff training [17].

Our case indicates that it is prudent for healthcare staff supervising adults with dementia to undertake an osteoporosis medication review, particularly if new swallowing issues or retention of food or tablets in oral tissues are noted by carers.

## 4. Limitations

This is a single case, and causality is inferred from temporal association and resolution on withdrawal rather than a histopathological diagnosis or rechallenge. Some potentially relevant baseline information was unavailable or not documented. The absence of these data limits the generalization of our conclusions to all older adults on oral bisphosphonate therapy.

## 5. Conclusions

This case highlights important prescribing considerations, namely that an older person’s ability to swallow and safely administer an oral bisphosphonate should be assessed before treatment is initiated and reassessed if cognition, swallowing function or functional status declines.

Amongst older adults who cannot reliably comply with administration requirements, continued prescribing of an oral bisphosphonate may expose them to avoidable mucosal and oesophageal injury. New oral pain, reduced oral intake, unexplained ulceration or facial swelling should prompt an examination of the oral cavity and medication review. In suspected cases of bisphosphonate-associated oral ulceration, withdrawal of therapy should be considered alongside osteoporosis risk assessment. Alternative antiresorptive strategies may be appropriate when oral treatment is impractical.

Medication safety in frail, older adults must encompass not just a drug dose, but the formulation, swallowing ability, cognitive status, patient positioning, supervision and practical ability to administer the drug.

## Data Availability

The original contributions presented in this study are included in the article. Further inquiries can be directed to the corresponding author.
